# Gastritis cystica profunda in an unoperated stomach mimicking a pyloric submucosal tumor and causing anorexia: A case report and literature review

**DOI:** 10.1097/MD.0000000000037652

**Published:** 2024-03-29

**Authors:** Seito Shimizu, Hitoshi Hara, Yasuhide Muto, Tomoki Kido, Ryohei Miyata

**Affiliations:** a Department of Surgery, Social Welfare Organization Saiseikai Imperial Gift Foundation Inc., Saiseikai Kazo Hospital, Kazo, Japan.

**Keywords:** case report, gastritis cystica profunda, submucosal cystic lesion of stomach, unoperated stomach

## Abstract

**Background:**

Gastritis cystica profunda (GCP), commonly observed in remnant gastric anastomosis, is associated with developing gastric cancer.

**Case:**

This case report describes a patient with GCP in a previously unoperated stomach that mimicked a pyloric submucosal tumor and caused anorexia, which is rare in clinical practice.

**Patient concerns:**

A 72-year-old woman presented with loss of appetite and weight.

**Diagnoses:**

Gastroscopy detected a 20 mm diameter submucosal tumor near the pylorus. Computed tomography and magnetic resonance imaging identified a cystic lesion, unlike a usual submucosal tumor in the stomach. The diagnosis was difficult, even with endoscopic ultrasound-guided fine-needle aspiration.

**Interventions:**

Surgery was performed for diagnosis and treatment. The lesion was resected using a submucosal dissection technique after an incision of the gastric wall during open laparotomy. Histopathological examination confirmed the diagnosis of GCP and revealed no dysplasia or cancer.

**Outcomes:**

Anorexia resolved after the surgery. Residual or recurrent lesions were not detected during follow-up examinations performed 1 year after surgery.

**Lessons:**

GCP occurring in a previously unoperated stomach as a macroscopic lesion like a submucosal tumor causing some symptoms is rare. GCP is associated with a risk of developing cancer. Therefore, careful evaluation and management during treatment are required.

## 1. Introduction

Gastritis cystica profunda (GCP) is a cystic lesion lined with hyperplastic gastric epithelium characterized by dilatation of the gastric glands extending into the mucosa and submucosal layer of the stomach. GCP is commonly observed in remnant gastric anastomosis and is considered to be associated with the development of remnant gastric cancer.^[1]^ GCP development in unoperated stomachs is rare. Mucosal injury through chronic inflammation has been suggested to cause GCP as a hyperplastic change, even in unoperated stomachs.^[2]^

Most cases of GCP in unoperated stomachs are pathologically identified within resected specimens obtained during gastric cancer surgeries and are considered to be associated with the development of gastric cancer. In the reported cases of GCP without the development of adenocarcinoma in the unoperated stomach, GCP presented with upper gastroenterological symptoms such as upper abdominal pain, acid reflux, nausea, anorexia, and bleeding; however, some patients might be asymptomatic.^[3]^ Although it is rare for GCP to present as a macroscopic lesion and cause any symptoms, we found that GCP should be considered in the case of cystic lesions mimicking a submucosal tumor in the stomach. Careful evaluation is required during treatment because there is a chance of cancer development in the GCP.

In this report, we present a unique clinical case of a patient with GCP without cancerous development in a previously unoperated stomach that mimicked a pyloric submucosal tumor and caused anorexia.

## 2. Case presentation

The patient was a 72-year-old woman with a medical history of atrophic gastritis, variant angina, uterine myomectomy, and *Helicobacter pylori* infection (5 years since eradication). For 2 months, she experienced appetite loss and epigastric discomfort. Subsequently, she sought medical attention at a nearby clinic and was prescribed proton pump inhibitors; however, her symptoms did not improve. She occasionally visited the emergency department because of severe nausea. Notably, she experienced weight loss due to a decreased appetite. The patient was referred to our hospital because of persistent symptoms.

**Figure 1.**
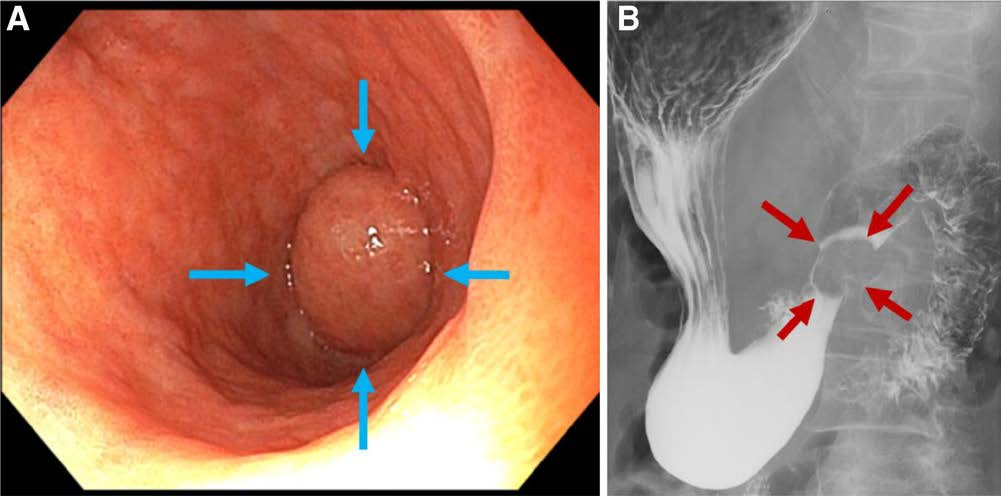
(A) Gastroscopy (blue arrows) and (B) gastrointestinal contrast series (red arrows) revealed an elevated 20 mm lesion near the pylorus similar to a submucosal tumor.

**Figure 2.**
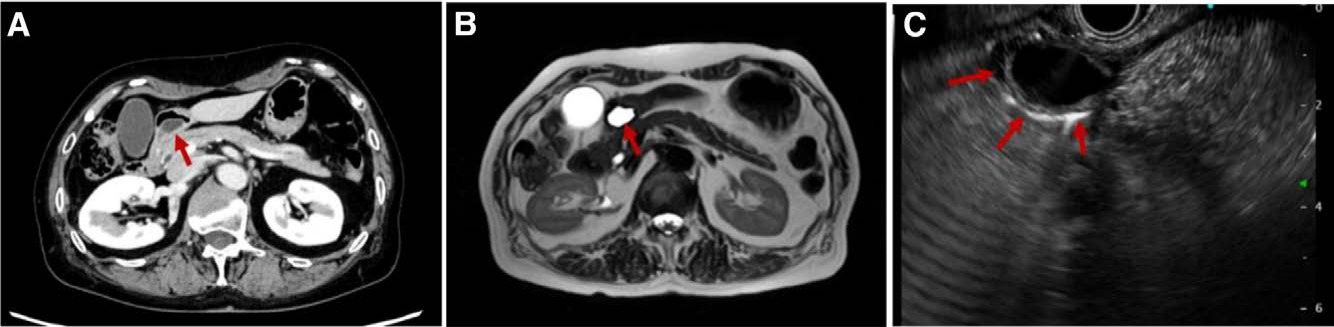
(A) Computed tomography (red arrow) and (B) magnetic resonance imaging (red arrow) revealed that the elevated lesion is a submucosal cystic lesion. (C) EUS (red arrows) showed a cystic lesion in the submucosal layer, but a pathological diagnosis was not made because there was insufficient tissue in the combined EUS-FNA. EUS = endoscopic ultrasound, EUS-FNA = endoscopic ultrasound-guided fine needle aspiration.

Gastroscopy and an upper gastrointestinal contrast series revealed an elevated lesion, 20 mm in size, near the pylorus, similar to a submucosal tumor (SMT) (Fig. 1A, B). Computed tomography, magnetic resonance imaging, and endoscopic ultrasound (EUS) revealed that the elevated lesion was a submucosal cyst (Fig. 2A–C). Differential diagnoses included a simple cyst, gastrointestinal stromal tumor, including leiomyoma or neuroma as an atypical submucosal tumor, lymphangioma, cyst-forming ectopic pancreas with or without cancer, GCP, and submucosal tumor-like gastric cancer developed from GCP, among others. Diagnosing the lesion pathologically was difficult, even when using EUS-guided fine needle aspiration (EUS-FNA). Thus, a decision was made to proceed with surgical intervention for diagnosis and treatment.

The submucosal cystic lesion was resected using a submucosal dissection technique after an incision of the gastric wall during open laparotomy. Intraoperative rapid pathological examination revealed a cystic lesion lined by columnar epithelium resembling a hyperplastic polyp without dysplastic or cancerous changes and no findings suggestive of malignancy. Therefore, gastrectomy with lymph node dissection was unnecessary.

**Figure 3.**
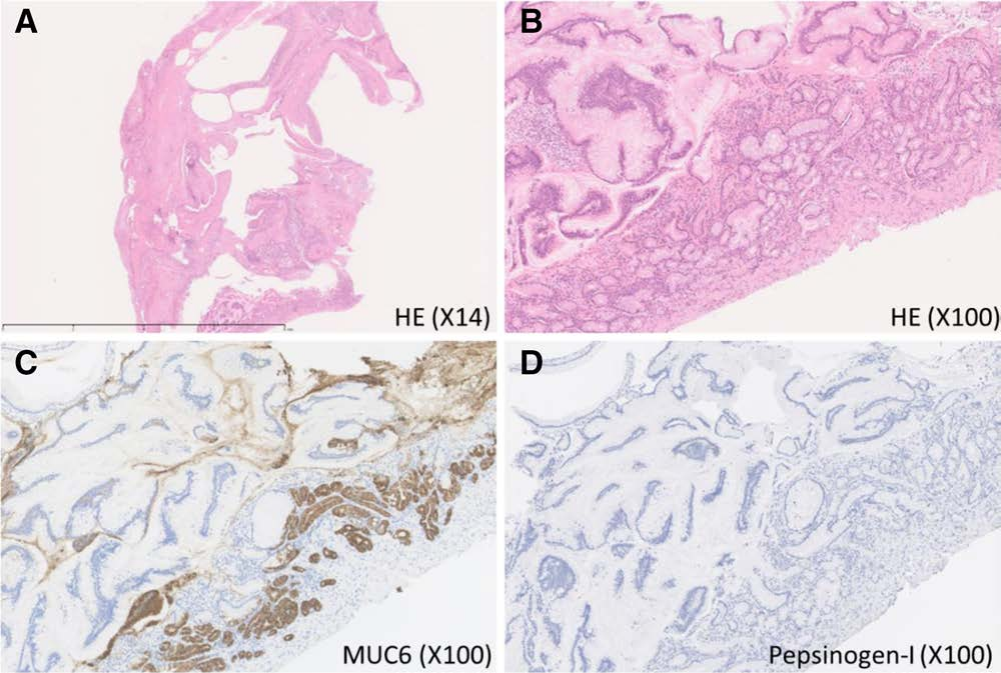
Histopathological findings. (A, B) Pathological findings showed multiple cystic structures lined by columnar epithelium in the submucosal layer. No findings of dysplastic or cancerous change were observed in these epithelial cells. The epithelium cells were (C) MUC6-positive and (D) Pepsinogen-1-negative in immunostaining, revealing the submucosal cystic lesion is lined by pyloric gland-like epithelium. Based on these findings, the cystic lesion was diagnosed as benign gastritis cystica profunda (A: 14×, B–D: 100× magnification) (A and B: hematoxylin and eosin staining, C and D: immunostaining).

GCP was pathologically diagnosed in a permanent specimen. In the permanent specimen, multiple cystic lesions lined with columnar epithelia appeared in the submucosal layer. The walls of the cystic lesion were MUC6-positive and pepsinogen1-negative on immunostaining, suggesting a submucosal cystic lesion lined with a pyloric gland-like epithelium. No dysplastic or cancerous changes were observed in the epithelial cells. Finally, the lesion was diagnosed as GCP without adenocarcinoma development (Fig. 3A–D).

The postoperative course was uneventful. Follow-up examinations performed 1 year after surgery did not detect any residual or recurrent lesions. Additionally, the patient’s weight increased owing to symptom improvement.

## 3. Discussion

In this clinical case report, we described a unique case of GCP without cancerous development in a previously unoperated stomach, mimicking a pyloric submucosal tumor causing anorexia and body weight loss. Gastrectomy with lymph node dissection was unnecessary because of the rapid intraoperative pathological examination result. The submucosal cystic lesion near the pylorus of the unoperated stomach was pathologically diagnosed as a benign GCP in the permanent specimen, and the patient’s symptoms improved after the surgery.

The polypoid mucosa of the gastric remnant’s gastroenterostomy site was initially termed “gastritis cystica polyposa” by Littler et al in 1972.^[1]^ The histopathological characteristics of gastritis cystica polyposa include hyperplasia of the gastric pit epithelium, atrophy of the corpus glands, the proliferation of pseudopyloric glands, and cystic dilation, and these cystic lesions were observed in the lamina propria and submucosal layers. Subsequently, Franzin et al reported the presence of cysts with histological features identical to gastritis cystica polyposa near ulcers and adenomas. They differentiated between lesions confined to the lamina propria and lesions located from the muscularis mucosae to the submucosal layer, proposing the latter to be “gastritis cystica profunda.”^[4]^

The occurrence of GCP at the anastomotic site after gastrectomy has been frequently reported.^[5]^ In patients with remnant stomach cancer, cancer development associated with GCP caused by chronic inflammation secondary to suturing or duodenal fluid reflux is postulated.^[4]^ A few reported cases of gastric cancer with a history of GCP in unoperated stomachs suggest that GCP may act as a potential origin for cancer development. However, definitive conclusions regarding the pathogenesis and treatment approach for GCP have not been reached.^[5]^

GCP is rare in unoperated stomachs and is primarily observed in pathological findings of resected gastric cancer specimens. Most GCP lesions in unoperated stomachs present as diffuse and small submucosal cystic lesions without a polypoid appearance. Therefore, GCP without malignant transformation is rarely identified as a macroscopic lesion through endoscopy or imaging diagnostics and rarely causes some symptoms.

Differential diagnoses of submucosal tumors of the stomach include gastrointestinal tumors, neuroendocrine tumors, sarcoma, malignant lymphoma, metastatic cancer, submucosal tumor-like gastric cancer, leiomyoma, neurofibroma, lipoma, inflammatory fibroid polyp, ectopic pancreas, lymphangioma, and simple cysts.^[6]^ However, cystic lesions are rare among these differential diagnoses. The conventional endoscopic forceps biopsy provides superficial mucosal information only. Therefore, the diagnosis of submucosal lesions is inferior. Although endoscopic ultrasound and EUS-FNA are highly valuable diagnostic tools for submucosal lesions,^[3]^ EUS-FNA has limitations in diagnosis because it pathologically evaluates only part of the lesion. Thus, for the definitive diagnosis of submucosal lesions, including the determination of malignant status, surgical excision is required.

Regarding the treatment of GCP, considering its potential premalignant nature, surgical excision with histopathological evaluation has been traditionally recommended.^[7]^ Okada et al reported a negative stance towards surgical excision.^[8]^ However, in certain cases, such as those with the possibility of malignant transformation during endoscopic surveillance,^[9]^ cases leading to compromised quality of life due to gastrointestinal symptoms such as abdominal pain and nausea, and cases resistant to conservative treatment, surgical resection should be considered.

We conducted a review of cases of GCP exhibiting some macroscopic lesions without malignant transformation in unoperated stomachs using the PubMed database. Nine cases were identified, including our case^[10–17]^ (Table 1). The median age was 57 years, with 3 cases observed in men and 6 cases in women (man-to-woman ratio of 1:2). The reported symptoms include abdominal fullness, abdominal pain, nausea/vomiting, anorexia, weight loss, and bleeding; however, one of the patients was asymptomatic and incidentally detected. The lesion locations were as follows: the gastric antrum in 4 patients, the gastric fundus in two, the pyloric region in two, and the gastric body in one. Endoscopic findings showed a submucosal mass or an SMT-like appearance in four, elevated nodular lesions in two, protrusion lesions in two, and small polyps in one. *Helicobacter pylori* infection was confirmed in 2 patients. Four (44.4%) patients had a history of gastritis. However, there have been only a few reports of GCP causing actual gastrointestinal obstruction. Upper abdominal symptoms such as appetite loss have been reported in patients with lesions near the pylorus or cardia. The treatments employed were distal gastrectomy in 3 patients, partial gastrectomy in two, endoscopic submucosal dissection in two, observation in one, and submucosal dissection with open laparotomy in one.

**Table 1:** Summary of reported cases of GCPs exhibiting some macroscopic lesions without malignant transformation in unoperated stomach.

Year	References	Age	Sex	HPI	Gastritis	Symptoms	Lesion location	Macroscopic findings	Treatment
2014	Wang et al	63	M	NR	Yes	Intermittent epigastric discomfort	Antrum	Elevated nodular lesion	Surgical resection
2014	Alkimawi et al	57	F	NR	No	Epigastric abdominal pain	Fundus	Small polyps	Surveillance
2014	Machicado et al	61	F	No	Yes	Dull epigastric pain	Gastric body	Submucosal mass	Partial gastrectomy
2015	Butt et al	38	F	Yes	Yes	Epigastric pain, fullness, nausea, vomiting, weight loss	Pylorus	Intramural mass	DG with duodenal fistula repair and GJ
2015	Yu et al	43	F	NR	No	Incidental	Antrum	Elevated nodular lesion	DG
2019	Deng et al	50	M	NR	No	Fullness, nausea	Fundus	Protrusion lesion	ESD
2020	Du et al	43	F	NR	No	Intermittent epigastric discomfort	Antrum	Submucosal mass	DG
2023	Cao et al	58	M	NR	No	Abdominal pain	Antrum	Protrusion lesion	ESD
2023	Our case	72	F	Yes	Yes	Anorexia, appetite loss, wight loss	Pylorus	SMT-like elevation	Submucosal dissection with open laparotomy

DG = distal gastrectomy, ESD = endoscopic submucosal dissection, F = female, GJ = gastrojejunostomy, HPI = helicobacter pylori infection, M = male, NR = not reported, SMT = submucosal tumor.

GCP is a conceivable diagnosis when differentiating cystic lesions presenting as gastric submucosal tumors. Because of GCP’s association with chronic gastritis and the potential for malignant changes, management strategies such as *Helicobacter pylori* testing and eradication, as well as vigilance for potential malignancy, should be considered by clinicians. Even in cases where histopathological examination indicates GCP without malignant transformation, it remains crucial to conduct follow-ups with an awareness of other potential latent GCP occurrences and the risk of carcinogenesis.

This case report had several limitations. The accuracy of diagnosis using rapid intraoperative pathological examinations is limited. If intraoperative rapid pathological examination fails to identify malignancy, but pathological diagnosis of the permanent specimen reveals malignancy, secondary surgery, which is gastrectomy with lymph node dissection, is required.

## 4. Conclusions

We presented a case of a patient with GCP in an unoperated stomach confirmed as an SMT-like submucosal cystic lesion leading to anorexia and weight loss but without concomitant carcinogenesis. Because GCP, with or without malignancy, is a challenging condition for preoperative diagnosis, surgical resection of GCP should be considered in cases of severe symptoms or resistance to conservative treatment. Our case emphasizes the importance of careful evaluation and follow-up in managing GCP.

## Acknowledgements

The authors thank Dr Akina Kawahara for her support with the pathological diagnosis, Editage (www.editage.jp) for English language editing, and Ms. Satoko Fukaya, who works at our hospital library.

## Author contributions

**Conceptualization:** Seito Shimizu, Hitoshi Hara.

**Data curation:** Seito Shimizu.

**Investigation:** Seito Shimizu, Hitoshi Hara.

**Project administration:** Hitoshi Hara.

**Resources:** Seito Shimizu, Hitoshi Hara, Yasuhide Muto, Tomoki Kido, Ryohei Miyata.

**Supervision:** Ryohei Miyata.

**Visualization:** Seito Shimizu.

**Writing – original draft:** Seito Shimizu.

**Writing – review & editing:** Hitoshi Hara.
